# Supplementary material for: Inkjet printing-based volumetric display projecting multiple full-colour 2D patterns
Source: Sci Rep. 2017 Apr 13;7:46511. doi: 10.1038/srep46511 (PMC5390278; doi:10.1038/srep46511)
Supplement: Supplementary Information [file srep46511-s1.pdf]

Supplementary Information for:

Inkjet printing-based volumetric display projecting multiple full-colour 2D patterns

Ryuji Hirayama[1,2,*], Tomotaka Suzuki[1], Tomoyoshi Shimobaba[1], Atsushi Shiraki[3], Makoto Naruse[4], Hirotaka Nakayama[5], Takashi Kakue[1] and Tomoyoshi Ito[1]

[1]Graduate School of Engineering, Chiba University, Chiba University, 1-33 Yayoi-cho, Inage-ku, Chiba 263-8522, Japan

[2]Research Fellow of the Japan Society for the Promotion of Science, 5-3-1 Kojimachi, Chiyoda-ku, Tokyo 102-0083, Japan

[3]Institute of Management and Information Technologies, Chiba University, 1-33 Yayoi-cho, Inage-ku, Chiba 263-8522, Japan

[4]Network System Research Institute, National Institute of Information and Communications Technology, 4-2-1 Nukui-kita, Koganei, Tokyo 184-8795, Japan

[5]Center for Computational Astrophysics, National Astronomical Observatory of Japan, 2-21-1 Osawa, Mitaka, Tokyo 181-8588, Japan

[*] hirayama@chiba-u.jp

Video Legend

Supplementary Video S1. Prototype of the inkjet printing-based volumetric display projecting three full-colour patterns. Each of the patterns can be obtained from each specific viewpoint.
